# A Very Long-acting Exatecan and Its Synergism with DNA Damage Response Inhibitors

**DOI:** 10.1158/2767-9764.CRC-22-0517

**Published:** 2023-05-24

**Authors:** Shaun D. Fontaine, Christopher W. Carreras, Ralph R. Reid, Gary W. Ashley, Daniel V. Santi

**Affiliations:** 1 ProLynx, San Francisco, California.

## Abstract

Exatecan (Exa) is a very potent inhibitor of topoisomerase I and anticancer agent. It has been intensively studied as a single agent, a large macromolecular conjugate and as the payload component of antigen-dependent antibody–drug conjugates. The current work describes an antigen-independent conjugate of Exa with polyethylene glycol (PEG) that slowly releases free Exa. Exa was conjugated to a 4-arm 40 kDa PEG through a β-eliminative cleavable linker. Pharmacokinetic studies in mice showed that the conjugate has an apparent circulating half-life of 12 hours, which reflects a composite of both the rate of renal elimination (half-life ∼18 hours) and release of Exa (half-life ∼40 hours). Remarkably, a single low dose of 10 μmol/kg PEG-Exa—only approximately 0.2 μmol/mouse—caused complete suppression of tumor growth of BRCA1-deficient MX-1 xenografts lasting over 40 days. A single low dose of 2.5 μmol/kg PEG-Exa administered with low but efficacious doses of the PARP inhibitor talazoparib showed strong synergy and caused significant tumor regression. Furthermore, the same low, single dose of PEG-Exa administered with the ATR inhibitor VX970 at doses of the DNA damage response inhibitor that do not affect tumor growth show high tumor regression, strong synergy, and synthetic lethality.

**Significance:**

A circulating conjugate that slowly releases Exa is described. It is efficacious after a single dose and synergistic with ATR and PARP inhibitors.

## Introduction

DNA-damaging agents—long-standing mainstays of cancer chemotherapy—have taken on added importance as agents that enhance DNA damage response (DDR) defects and inhibitors in tumors. DNA damage in the face of impaired ability to repair damaged DNA can result in selective toxicity and synthetic lethality (1). An important target to cause-specific DNA damage is the enzyme topoisomerase I (TOP1), which is inhibited by camptothecin and its analogs (2, 3). During DNA replication, TOP1 relaxes supercoiled DNA by forming a covalent phosphodiester with DNA, permitting the broken strand to rotate around and relax the TOP1-bound DNA, and then reversing covalent binding to religate DNA. In the presence of an effective TOP1 inhibitor (TOP1i) such as a camptothecin analog, the covalent TOP1i–DNA complexes are specifically trapped, forming a TOP1 cleavage complex (TOP1cc). Subsequently, single and double DNA strand breaks form that lead to cell death if unrepaired. Because the TOP1cc rapidly reverses upon drug removal, presence of the TOP1i must be maintained to translate into DNA damage.

One of the attractive features of TOP1 as a cancer chemotherapy target is the possibility of predicting patient response through the use of biomarkers. Homologous recombination deficiency and expression of the putative DNA/RNA helicase Schlafen 11 (SLFN11) have recently been shown to serve as predictive biomarkers for tumor sensitivity to TOP1is (4–7). Indeed, Coussy and colleagues (7) demonstrated that patient-derived triple-negative breast cancer (TNBC) xenografts with BRCAness or high SLFN11 showed 50% and 75% response rates to the TOP1i irinotecan (CPT-11), respectively, and xenografts containing both biomarkers were 100% responsive. Translation of these preclinical results to humans would have major impact in personalized, targeted therapy. We have an ongoing program aimed at developing best-in-class TOP1i for use in the setting of DDR deficiencies and inhibitors.

SN-38—the active metabolite of the anticancer agent irinotecan, CPT-11—is a potent TOP1i. However, CPT-11 is a less than ideal prodrug: it is extensively metabolized in the liver and is notoriously variable in its pharmacokinetics, pharmacodynamics, and toxicities (8). The SN-38 metabolite formed from CPT-11 in the liver also undergoes glucuronide-mediated enterohepatic metabolism that can lead to severe gastrointestinal toxicity. These multiple metabolic and transport processes lead to high interpatient variability of CPT-11 efficacy and toxicity which has stimulated efforts in developing more suitable prodrugs of TOP1is.


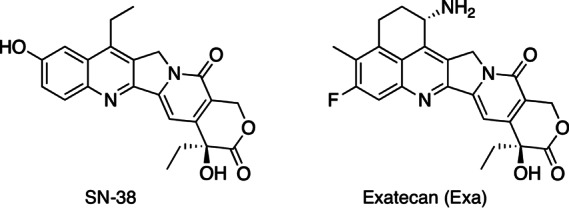


Another TOP1i of high current interest is the camptothecin analog exatecan (Exa), an even more potent TOP1 trapping agent/inhibitor than SN-38 (5, 9); for a comprehensive review, see ref. 10. As a single agent, Exa progressed as far as phase III clinical trials before its development was discontinued because of dose-limiting side effects, and the absence of therapeutic benefits in combination with gemcitabine (11).

One of the limitations of current small-molecule TOP1i is their relatively short half-lives—the SN-38 formed from CPT-11 has an apparent t_1/2_ of approximately 12 hours (8) and Exa has a t_1/2_ of only approximately 10 hours in humans (12). As described above, effective suppression of TOP1 to cause DNA damage requires continuous presence of the inhibitor during S-phase of the cell cycle and inhibition of TOP1 is rapidly reversed upon drug withdrawal. Hence, the short half-lives of these TOP1i are suboptimal for drug efficacy, and extended-release forms of these agents are expected to show improved efficacy.

An approach toward obtaining longer-lived TOP1i that actively target specific tumors is to incorporate them as payloads in antibody–drug conjugates (ADC). Indeed, three ADCs containing TOP1is—the Trop2-directed sacituzumab govitecan (Trodelvy) delivering SN-38, the HER2-targeted trastuzumab deruxtecan (Enhertu), and Trop2-directed datopotamab deruxtecan delivering a close derivative of Exa—show impressive results in the clinic and have been approved (13). However, most tumors do not have the Trop2 or HER2 surface antigens recognized by these ADCs, and are thus not suitable targets for these tumor-targeted agents. Likewise, ADCs cannot specifically target subsets of a tumor that differs from others in a TOP1i DDR defect, such as BRCA or ATM deficiency. Thus, a need remains to develop long-acting, tumor-agnostic TOP1i prodrugs that do not require the presence of specific tumor antigens to express their antitumor effects.

Another approach toward obtaining long-lived TOP1i has been to develop passively targeted antigen-independent prodrugs in which the TOP1i is attached to a tumor-accumulating macromolecular carrier by a releasable linker. Depending on the linker used the TOP1i may be slowly released from the prodrug systemically and/or after penetrating the tumor by the EPR effect. For example, DE-310 is a conjugate of a large 340 kDa CM-dextran polyalcohol carrier covalently linked to Exa by a cathepsin-sensitive peptidyl spacer (14). The conjugate has a long systemic t_1/2_ (15, 16) and passively accumulates in xenografts where, after cell internalization, lysosomal cathepsins cleave the linker to release Exa (17). Although DE-310 suppressed growth of xenografts for long periods (14), accumulation of the prodrug in human tumors was not observed (18) and subsequent efforts with Exa were apparently redirected toward its use as a payload in ADCs. It would be advantageous to have a TOP1i prodrug that can passively target tumors but does not mandate internalization and proteolysis for activity.

**SCHEME 1:**
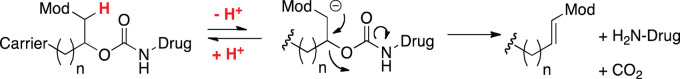


We have had an interest in developing a general approach for half-life extension of therapeutics in which a drug is covalently tethered to a long-lived carrier by a linker that slowly cleaves by β-elimination to release the drug (Scheme 1; ref. 19). The cleavage rate, k_1_, is determined by the nature of an electron-withdrawing “modulator” (Mod) that controls the acidity of the adjacent C–H bond, and is unaffected by enzymes or general acid/base catalysts. A carrier used for β-eliminative linkers is often a long-lived circulating macromolecule—such as high molecular weight polyethylene glycol (PEG). For this purpose, 4-arm PEG_40kDa_—a 15-nm-diameter nanocarrier—may be optimal because smaller PEGs have shorter half-lives, while larger PEGs all show similar elimination rates. The prodrug is usually eliminated with a t_1/2_ similar to that for carrier, and the apparent t_1/2_ of the released drug is usually similar to the t_1/2_ of the prodrug. Furthermore, tumor accumulation occurs because of the long circulating t_1/2_ and near-ideal size and shape of the 15-nm-diameter nanocarriers to penetrate large pores of tumor vasculature (20, 21).

We used this technology to prepare the passively-targeted PEG_40kDa_-SN-38 conjugates PLX038 and PLX038A that spontaneously slowly release the potent TOP1i SN-38 by a β-elimination reaction (22, 23). The favorable properties of these prodrugs are (i) a long t_1/2_ of released SN-38 approximating the t_1/2_ of the PEG_40kDa_ carrier with concomitant benefits of low *C*_max_ and prolonged exposure, (ii) high accumulation and retention in tumors (20, 22), and (iii) a high therapeutic effect in tumors with DDR defects and in combination with DDR inhibitors (22). Hence, PLX038 provides a prolonged duration of DNA damage to achieve synthetic lethality of DNA repair deficient or inhibited tumors.

The objective of the current work was to design an efficacious long-acting PEG-Exa prodrug analogous to PLX038 that slowly releases Exa but—unlike ADCs with a TOP1i payload—is not limited to cancers expressing high levels of a specific tumor antigen. Here, we (i) describe the synthesis and characterization of a PEG-Exa prodrug, (ii) ascertain its pharmacokinetics in mice, and (iii) determine antitumor effects of the conjugate in mouse xenografts. We report that a single injection of the PEG_40kDa_-Exa provides effective and long-lasting antitumor effects in a BRCA1-deficient mouse xenograft and shows synergy with PARPi talazoparib (TLZ) and the ATR inhibitor (ATRi) VX970.

## Materials and Methods

### General Methods

1. **Materials**. PEG_40kDa_-[NH_2_]_4_ was purchased from NOF America (Sunbright PTE-400PA). PEG-(CO_2_H)_4_ was purchased from SINOPEG (6020700415). Exa was purchased from BOC Sciences (B0084-060840). All other commercially available chemicals were purchased as reagent grade and used without further purification.
2. **Equipment**. Unless otherwise specified, high-performance liquid chromatography (HPLC) analyses were performed on a Shimadzu LC-20AD HPLC system equipped with an SPD-M20A diode array detector and RF-10AXL fluorescence detector fitted with a Phenomenex Jupiter 5 μmol/L C18 column (300 Å, 150 × 4.6 mm). Synthetic reactions were monitored using an HPLC mobile phase of H_2_O/0.1% trifluoroacetic acid (TFA) and MeCN/0.1% TFA. Preparative HPLC was performed on a Shimadzu LC-20AP system equipped with an SPD-20A UV-vis detector, FRC-10A fraction collector, and Phenomenex Jupiter C18 column (300 Å, 150 × 21.2 mm). Exa and PEG-Exa conjugates 3A and 3B were monitored by UV absorbance at 370 nm and using fluorescence (λ_ex_ 365 nm, λ_em_ 450 nm). UV-vis data were acquired on a Hewlett Packard 8453 UV-vis spectrometer. ES^+^/TOF mass spectra were obtained at the University of California, San Francisco Small Molecule Discovery Center core facility using a Waters Xevo G equipped with an Aquity solvent delivery system.

### Synthesis

**1. Releasable PEG-Exa** 3A

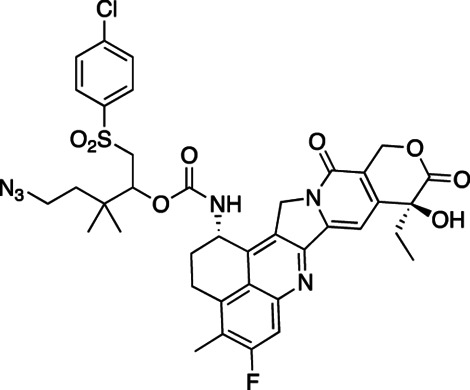

**5-azido-1-((4-chlorophenyl)sulfonyl)-3,3-dimethylpentan-2-yl exatecan carbamate (2A).** A solution of exatecan mesylate (510 mg, 0.96 mmol, 1 equiv), iPr_2_NEt (184 μL, 1.06 mmol, 1.1 equiv) in DMF (6.4 mL) was treated with 5-azido-1-(4-chloro-phenylsulfonyl)-3,3-dimethyl-2-pentyl succinimidyl carbonate (682 mg, 1.44 mmol, 1.5 equiv) HSC (24). The reaction mixture was stirred at ambient temperature for 4 hours. The reaction mixture was diluted with EtOAc (50 mL) and 5% KHSO_4_ (25 mL). The aqueous phase was separated and extracted with EtOAc (3 × 25 mL). The combined organic phases were washed with brine, dried over MgSO_4_, filtered, and concentrated. Purification via column chromatography (80 g silica gel; acetone/dichloromethane) afforded 689 mg (0.87 mmol, 91% yield).
LC/MS (ESI) m/z calcd for C38H39ClFN6O8S^+^: 793.21; found: 793.32.
Purity was determined by C18 HPLC monitored at 363 nm: 96% [0%–100%B, RT = 11.02, 11.11 minutes (diastereomers)].

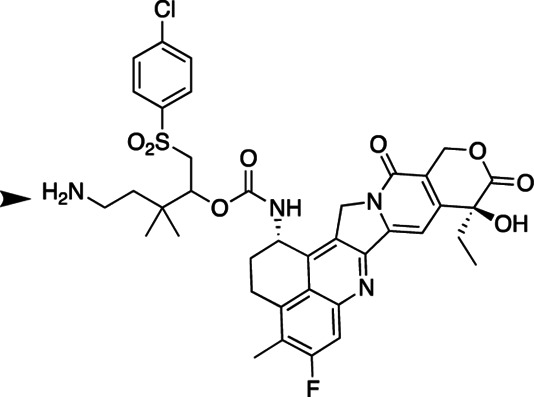

**5-amino-1-((4-chlorophenyl)sulfonyl)-3,3-dimethylpentan-2-yl exatecan carbamate trifluoroacetate salt.** A 4-mL glass vial with screw cap was charged with 2A (79 mg, 100 μmol, 1 equiv, final concentration 0.1 mol/L), THF (0.5 mL), AcOH (14 μL, 250 μmol, 2.5 equiv), and a solution of Me_3_P (1 mol/L THF, 0.5 mL, 500 μmol, 5 equiv). The reaction mixture was stirred at ambient temperature for 3 hours. C18 HPLC analysis showed complete conversion of 2A to the amine together with approximately 15% of an unknown exatecan-containing byproduct. The reaction was dried, and the crude material was redissolved in DMF, filtered through a 0.2 μm syringe filter, and purified by preparative C18 HPLC (25%–70%B, H_2_O/MeCN/0.1% TFA) and dissolved in MeCN (2 mL) to afford a 28.5 mmol/L solution of the desired product (57 μmol, 57% yield).
LC/MS (ESI) m/z calcd for C38H41ClFN6O8S^+^: 767.22; found: 767.28.
C18 HPLC was determined by C18 HPLC monitored at 363 nm: 98% [two peaks, RT = 9.55, 9.88 minutes (diastereomers)].

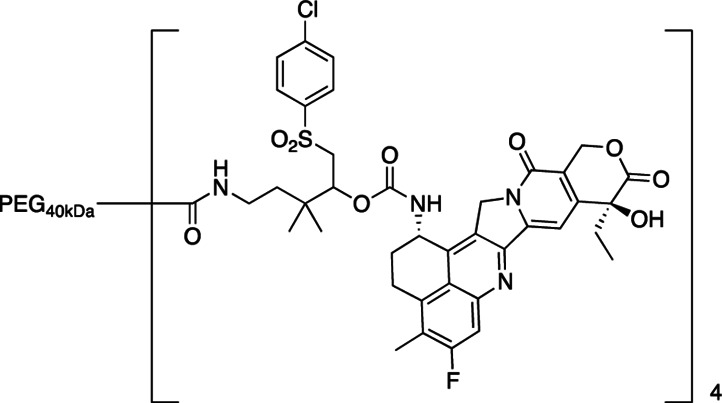

**PEG_40__kDa_-[exatecan]_4_ (Mod = 4-Cl-C_6_H_4_-SO_2_-) (3A**). A 50-mL round bottomed flask equipped with a stir bar, rubber septum, and nitrogen inlet was charged with a solution of 5-amino-1-((4-chlorophenyl)sulfonyl)-3,3-dimethylpentan-2-yl exatecan carbamate trifluoroacetate salt (76 mmol/L, 1.71 mL, 130 mmol, 1.3 equiv), 40-kDa PEG-(CO_2_H)_4_ (1.00 g, 100 mmol, 1 equiv), MeCN (8.5 mL), and iPr_2_NEt (180 μL, 1 mmol, 10 equiv). HATU (49 mg, 130 μmol, 1.3 equiv) was added as a solid in a single portion. The reaction mixture was stirred at ambient temperature for 1 hour, then concentrated to a yellow residue. The residue was dissolved in THF (10 mL) and added to 50% iPrOH/MTBE. The suspension was cooled on ice for 15 minutes and the resulting solids were collected by centrifugation and decanting of the supernatant. The solids were washed with 50% iPrOH/MTBE (3 × 50 mL) and 100% MTBE (3 × 50 mL) as above and dried under high vacuum until constant weight to afford 982 mg (92 μmol, 91% yield) as a white solid.
Purity was determined by C18 HPLC at 363 nm: 97% (0%–100%B, RT = 10.47 minutes).
**2. Stable PEG-Exa 3B**
Stable PEG-Exa was prepared in a similar fashion as above except 6-(Bocamino)hexyl N-(hydroxysuccinimidyl) carbonate was used as the linker.

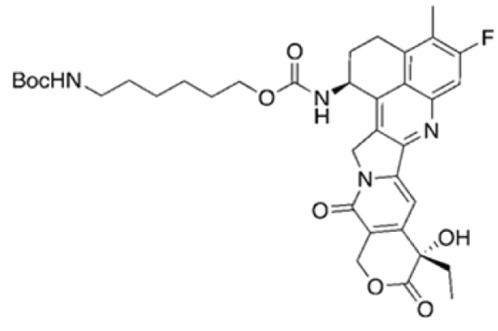

**6-(Boc-amino)hexyl exatecan carbamate** was prepared in a manner similar to that of compound 2A using 6-(Boc-amino)-1-hexyl succinimidyl carbonate to afford 319 mg (0.47 mmol, quantitative yield) of the desired product as a colorless solid.
LC/MS (ESI) m/z calcd for C36H44FN4O8^+^: 679.31; found: 679.34
Purity was determined by C18 HPLC at 363 nm: 99% (0%–100%B, RT = 10.96 minutes).

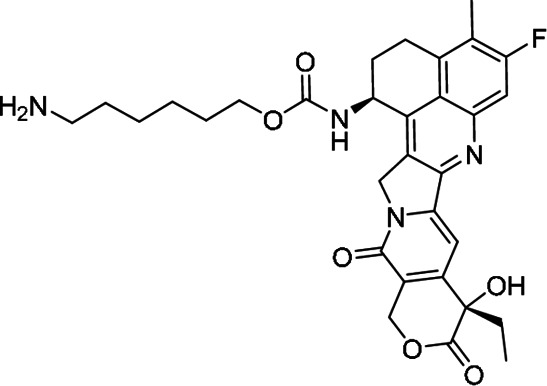

**6-aminohexyl exatecan carbamate trifluoroacetate salt**. A 20-mL scintillation vial equipped with a stir bar and screw cap was charged with a solution of 6-(Boc-amino)hexyl exatecan carbamate (319 mg, 0.47 mmol, 1.0 equiv) and dichloromethane (2 mL). The solution was cooled on ice while TFA was added rapidly dropwise via syringe. The reaction mixture was stirred on ice for 1 hour then concentrated to minimal volume and then from MeCN (5 mL) × 5. The crude product was dried under high vacuum for 1 hour and ambient temperature and used without further purification.
LC/MS (ESI) m/z calcd for C31H36FN4O6+: 579.25; found: 579.29.
Purity was determined by C18 HPLC at 363 nm: 96% (0%–100%B, RT = 8.86 minutes).

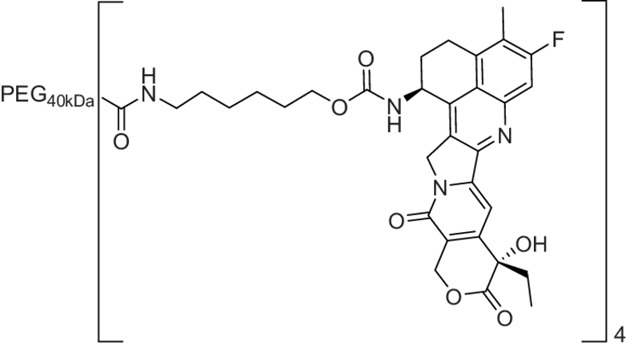

**Stable PEG-[Exatecan]_4_ (3B)** was prepared from the above aminohexyl exatecan carbamate in a manner similar to that of 3A. The crude product was dialyzed (SpectraPor 12-14,000 MWCO) against 500 mL 50% MeOH/H_2_O for 16 hours, then against 100% MeOH for 4 hours. The retentate was concentrated to a thick residue, dissolved in THF (8 mL) and added to 50 mL of 50% iPrOH/MTBE. The resulting suspension was cooled on ice for 15 minutes with periodic mixing by vortex. The resulting solids were collected by centrifugation and decanting of the supernatant. The solids were washed in a similar manner with 50% iPrOH/MTBE (3 × 50 mL) and 100% MTBE (3 × 50 mL) before drying under high vacuum for 3 hours to afford 1.3 g (0.12 mmol, 77% yield).
Purity was determined by C18 HPLC at 363 nm: 99% (0%–100%B, RT = 10.28 minutes).
Each molecule of 4-arm PEG conjugate contains 4 equivalents of exatecan. Doses are reported as μmol PEGylated exatecan/kg. For example, a dose of 15 μmol/kg contains 15 μmol Exa/kg on PEG.

### Pharmacokinetics

1. ***In vitro* cleavage of PEG-Exa 3A.** Solutions of 3A at pH 5.1 to 9.4 were kept at 37°C in an HPLC autosampler. At appropriate intervals, aliquots were injected on a C18 HPLC column and eluted with gradient of H_2_O and MeCN containing 0.1% TFA. Peak areas measured by UV-vis were fitted to a single-phase exponential equation to determine the half-life of linker cleavage.
2. **Pharmacokinetics in mice.** Pharmacokinetic studies were performed at Murigenics or Charles River Laboratories, using protocols approved by their Institutional Animal Care and Use Committees. Tumor xenograft studies were conducted at Murigenics. Solutions of stable (3B) and releasable (3A) Exa conjugates were prepared in isotonic acetate (pH 5), sterile filtered through a 0.2 μmol/L syringe filter, and Exa content was determined at A_370_ using ε = 19,000 M^−1^cm^−1^. The releasable conjugate 3A was administered intraperitoneally to male CD-1 mice (*N* = 4/group) at 40 or 15 μmol/kg, while the stable conjugate 3B was administered intraperitoneally or intravenously at 7.5 μmol/kg. Blood samples were obtained 1, 2, 4, 8, 12, 24, 48, 72, 96, and 120 hours after administration via serial tail-snip, immediately treated with 0.1 volume of 1 mol/L citrate (pH 4.5) solution and centrifuged to give plasma samples (15 μL) which were kept on ice during processing and frozen at −80°C until analysis.

Standard curves were generated by serial dilution of a solution of 3A, 3B or free Exa in mouse serum. A 10 μL aliquot of each standard or pharmacokinetic plasma sample was combined with an equal volume of mouse plasma containing 100 mmol/L citrate, pH 4.5, and 35 μmol/L ε−DNP-lysine internal standard. Acetonitrile (60 μL) was added to the mixture, vortexed for 10 seconds and clarified by centrifugation at 21,000 × *g*, 4°C, for 5 minutes. A portion of the supernatant (50 μL) was transferred to a 96-well plate and 150 μL of 0.33% acetic acid was added. The resulting samples (145 μL) were injected onto a Phenomenex Jupiter C18 300Å 5 μmol/L (150 × 4.6 mm) column heated to 40°C and eluted with H_2_O/MeCN/0.1% TFA (0%–100%B over 15 minutes). The conjugates and released Exa were monitored by UV absorbance at 370 nm. Low levels of Exa in plasma were monitored by fluorescence (λ_ex_ 365 nm; λ_em_ 450 nm).

Individual C versus t curves for each mouse were fit to a single exponential decay with 1/C^2^ weighting using data obtained from 24 to 120 hours. t_1/2_ values obtained for each mouse were combined to obtain a mean and SD for each group of animals.

### Antitumor Activity of PEG-Exa in the MX-1 Murine Xenograft

Female *scid* mice bearing MX-1 xenografts were prepared as reported previously. Animals were housed at Murigenics. MX-1 cells were obtained from Accegene and authenticated via 16-marker shorttandem repeat profile and shown to be negative for *Mycoplasma* contamination by PCR within 2 months prior to the start of these studies. Tumor volumes [caliper measurement: 0.5 × (length × width^2^)] and body weights were measured twice weekly. When tumors were approximately150 mm^3^_,_ mice (*N* = 6/group) received vehicle or a single intraperitoneal dose of approximately 15 mL/kg of a solution of PEG-(Exa)_4_ 3A in isotonic acetate buffer, pH 4.5 to deliver 2.5 to15 μmol/kg 3A. In a second experiment, female *scid* mice bearing MX-1 xenografts were prepared as above, and groups (*N* = 6) received either vehicle, a single intraperitoneal dose of 2.5 μmol/kg 3A, every day orally TLZ at 0.4 μmol/kg, orally VX-970 at 30 mg/kg (4 days on, 3 days off), a combination of 2.5 μmol/kg 3A and every day orally TLZ at 0.4 μmol/kg, or a combination of 2.5 μmol/kg 3A and orally VX-970 at 30 mg/kg (4 days on, 3 days off). VX-970 was prepared in 10% tocopherol-PEG1000 succinate/50 mmol/L NaOAc pH 5. TLZ was dissolved in 10% dimethylacetamide/6% Solutol/84% PBS at pH 7.4. TLZ and VX-970 were administered through the entire duration of the study.

Tumor growth (TG) and tumor growth inhibition (TGI) were calculated as described previously (25) using the percentage of the area under the tumor volume versus t curve (AUC) for the respective dose group in relation to the vehicle, such that TG = 100 × [AUC_treatment_/AUC_vehicle_] and TGI = 100 × [1 − (AUC_treatment_)/(AUC_vehicle_)].

To assess the interaction of the drug combination, we used an additivity index determined as TG_calc_/TG_obsd_ where TGExa_obsd_ and TG_drug_ are the observed tumor growth in the presence of individual drugs and TG_calc_ is the product of TG_obsd_ values of the individual drugs. Here, an index <1 indicates infra-additive, an index of 1 indicates additivity and an index >1 indicates a supra-additive or synergistic interaction. Supplementary Table S3 shows the values for synergy for 3A and the ATRi VX970.

### Data Availability

Data were generated by the authors and available on request.

## Results

### Synthesis and *In Vitro* Characterization of 4-arm PEG_40kDa_-Exa Conjugates

**SCHEME 2:**
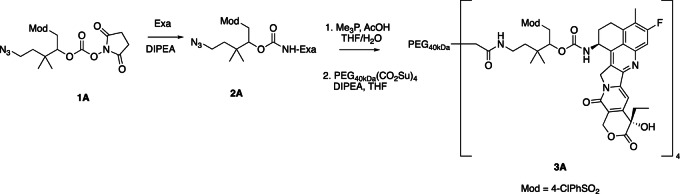


**FIGURE 1:**
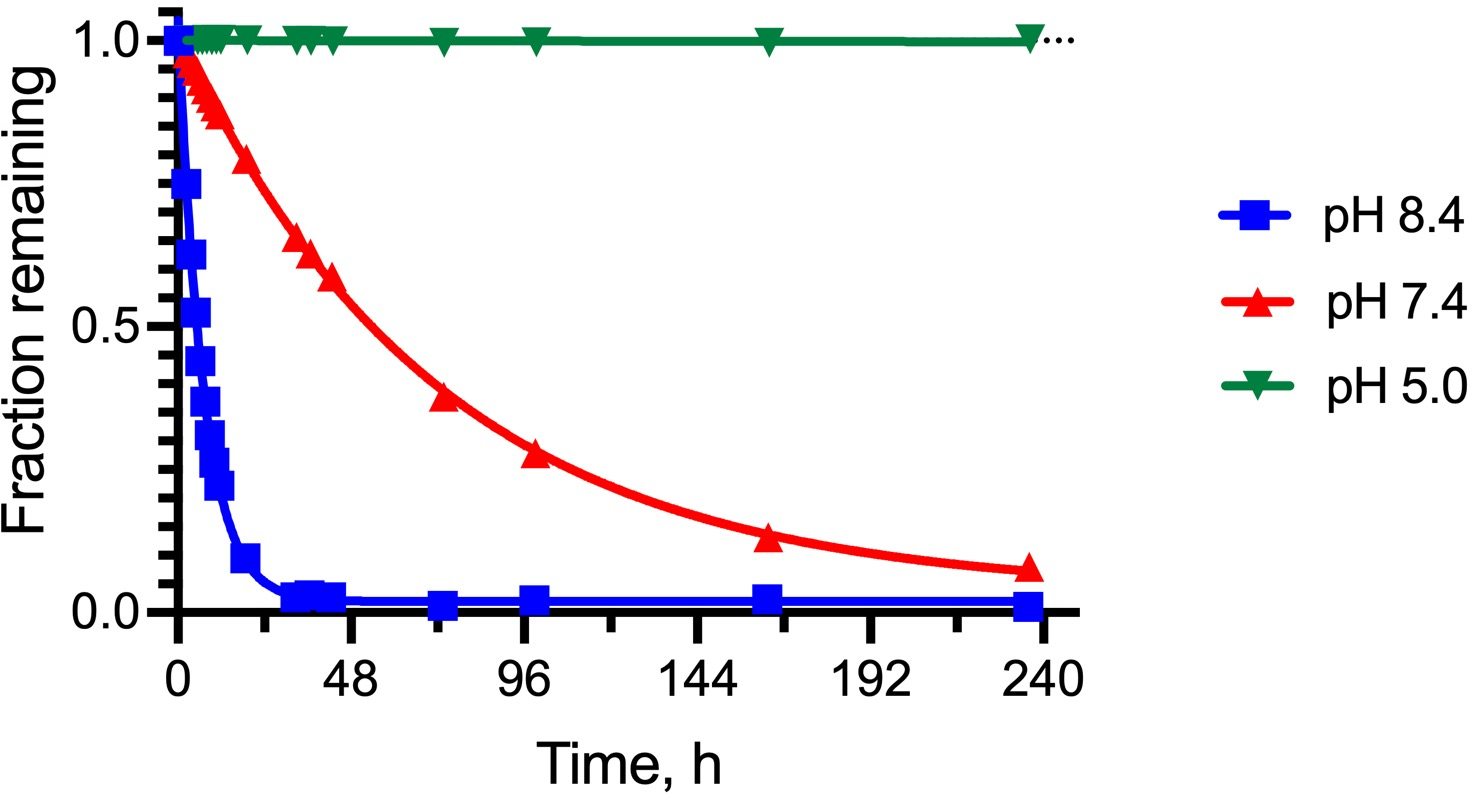
PEG-Exa remaining over time at 37°C at pH 5.0–8.4.

The releasable PEG_40kDa_-Exa conjugate 3A and its stable counterpart 3B were prepared by methods used for analogous carbamate conjugates (Scheme 2; refs. 19, 23). Appropriately-protected linker succinimidyl carbonates—1A for 3A and 6-tBoc-amino hexanoyl HSI carbonate for 3B—were coupled to Exa via carbamates and then deprotected to give amino-linker-Exa conjugates; the amino-linker-Exa were then coupled to 4-arm PEG_40kDa_(CO_2_H)_4_ using HATU to give the amide-linked conjugates 3A and 3B. The *in vitro* rates of Exa release from 3A at 37°C indicated a hydroxide-catalyzed reaction with k_OH_ 0.012 h^−1^ between pH 7.4 and 9.0 and a cleavage t_1/2_ of approximately 60 hours at pH 7.4 (Fig. 1; Supplementary Table S1); here, HPLC showed quantitative conversion of 3A to Exa. At pH 5.0, 99% of the conjugate 3A remained at 10 days. Taken together, the data confirm a base-catalyzed β-elimination of PEG_40kDa_-Exa 3A to give PEG_40kDa_ and free Exa, and demonstrate that the releasable conjugate 3A is sufficiently stable to allow storage at pH 5.

### Pharmacokinetics of PEG_40kDa_-Exa

**FIGURE 2:**
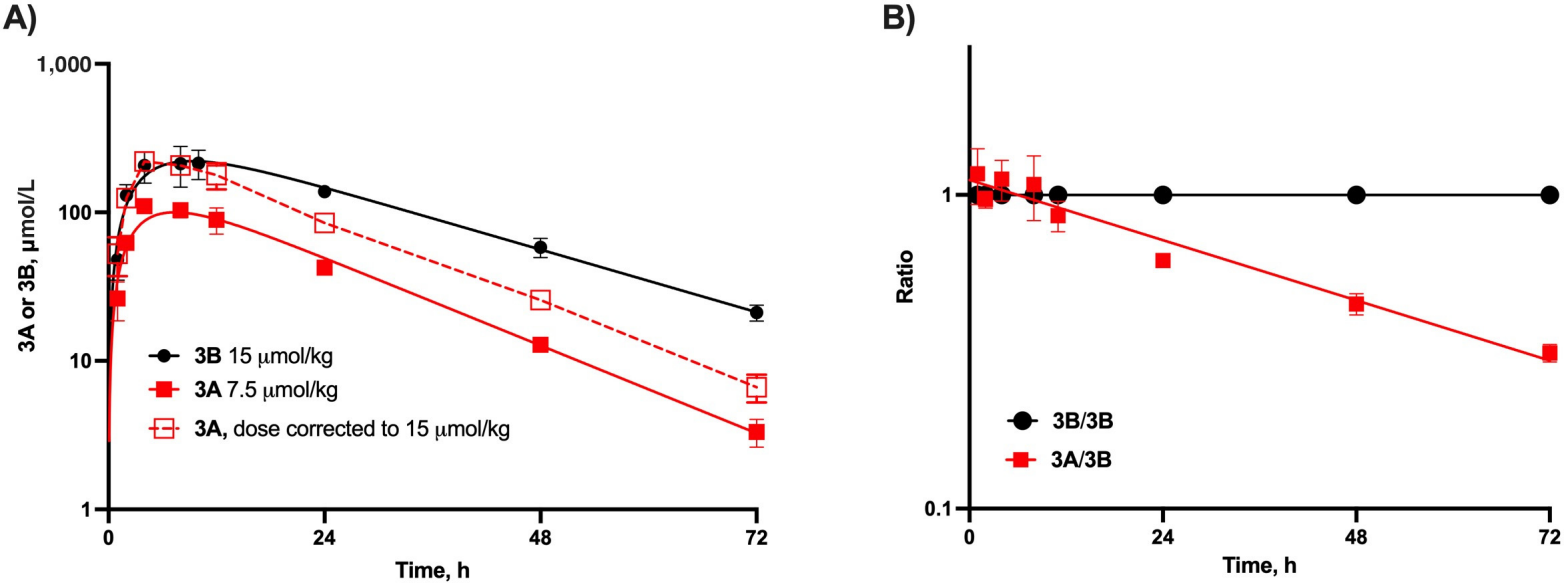
Pharmacokinetic profile of PEG-Exa conjugates 3A and 3B. **A,** C versus t plots after intraperitoneal administration of 7.5 μmol/kg 3A and 15 μmol/kg 3B to mice (*n* = 4/group); data for 3A normalized to 15 μmol/kg are also shown. Average C versus t curves for each group of mice were fit to a model of absorption followed by elimination [C = C_0_(exp(−k_elim_t) − exp(−k_abs_t)] with 1/C^2^ weighting. **B,** Replot of the data in A as the 3B/3B and 3A/3B ratios where slope of the latter is the rate constant for Exa release, k_1_, from 3A (19).

Figure 2 shows the C versus t plot after intraperitoneal injection of releasable PEG_40kDa_-Exa 3A and stable PEG_40kDa_-Exa 3B in the mouse; Table 1 shows the pharmacokinetic parameters determined from the experiments as well as dose normalized values calculated as AUC/dose and *C*_max_/dose. After an absorption phase, PEG_40kDa_-Exa 3A shows an apparent elimination t_1/2,β_ of approximately 12 hours while stable PEG-Exa 3B shows an apparent elimination t_1/2_ of approximately 18 hours, similar to the elimination rate of a typical PEG_40kDa_ in the mouse (19). The rate constant for Exa release from 3A, k_1_, can be determined using the equation k_1_ = k_3_ − k_β_ (19) where k_3_ is the terminal elimination rate constant for the stable surrogate 3B and k_β_ is the terminal elimination rate constant for 3A. From this, we estimate a t_1/2_ of approximately 40 hours for release of Exa from 3A *in vivo*, somewhat faster than the *in vitro* cleavage rate at pH 7.4, 37°C. In an experiment using a high 40 μmol/kg 3A to increase assay sensitivity of Exa analysis (Supplementary Fig. S1; Supplementary Table S2) the released free Exa showed a t_1/2_ of approximately 25 hours.

**TABLE 1:** Pharmacokinetic parameters of 3A and 3B in the mouse^a^

	PEG-Exa (3B)	PEG-Exa (3B)	PEG-Exa (3A)
**PEG-Exa conjugates**			
Dose, μmol/kg	15	15	7.5
Route of administration	Intraperitoneal	Intravenous	Intraperitoneal
t_1/2,α_, hours	3.0 ± 0.5		2.6 ± 0.3
t_1/2,β_, hours	17.2 ± 1.2	18.3 ± 0.6	12.2 ± 0.5
t_max_, hours	10		7
t_1/2_ drug release, hours			40.8 ± 9.1
*C*_max_, μmol/L	214 ± 24	307 ± 41	110 ± 4
*C*_max_/dose, kg/L	14.3 ± 3.2	20.5 ± 5.4	14.7 ± 1.1
AUC_∞_, μmol/L•hours	8,140 ± 595	6,930 ± 1477	1,650 ± 116
AUC_∞_/dose, kg•hours/L	543 ± 40	462 ± 99	220 ± 16
%F	118 ± 24%	100%	NA
C_0_, μmol/L	391 ± 56		193 ± 21
C_0,_ μmol/L/dose, kg/L	26 ± 4		25 ± 3
*V*_d_, L/kg		0.05	
**Released Exa**			
*C*_max_, μmol/L	NA	NA	0.028 ± 0.009
*C*_max_/dose, kg/L	NA	NA	0.004 ± 0.001

Abbreviation: NA, not applicable.

^a^Values for mean ± SEM derived from the data shown in Fig. 1. Error values are symmetrical SEs generated by GraphPad Prism 9.4.1.

### Tumor Inhibition by PEG-Exa

**FIGURE 3:**
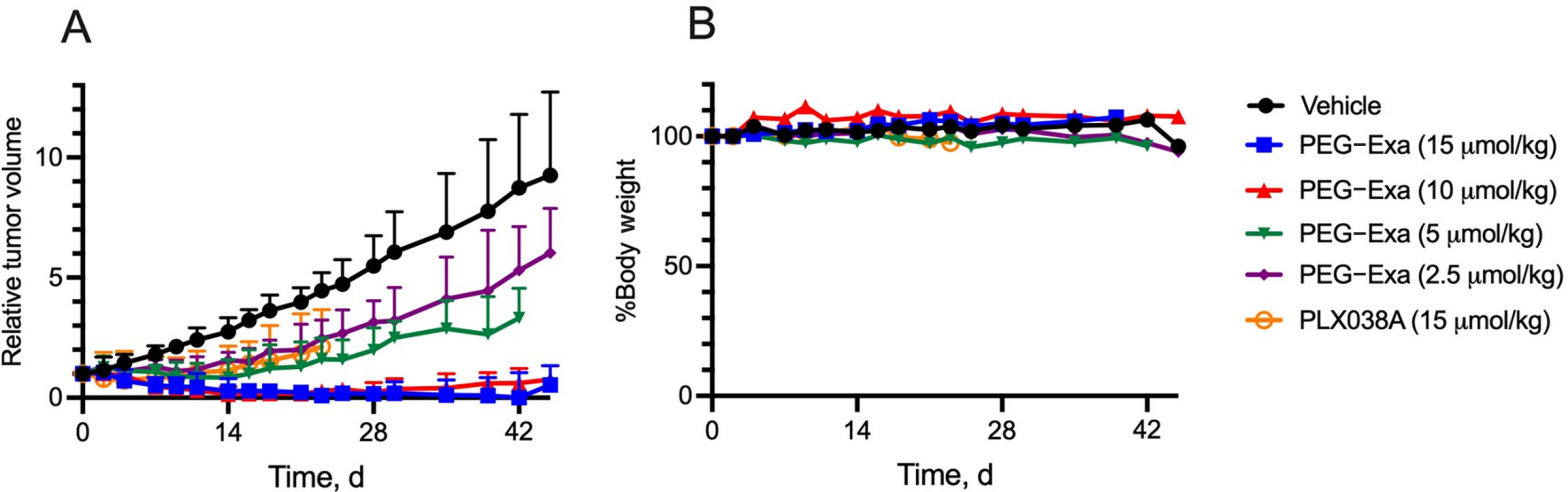
Treatment of MX-1 xenografts (N-6/group) with single doses of PEG-Exa 3A and PLX038A. **A,** Median relative tumor volume ± interquartile range versus t. The starting tumor volume for all groups was 129 ± 38 mm^3^. *P* values at 21 days from one-way ANOVA analysis versus vehicle were: 0.008 (PLX038A), 0.01 (2.5 μmol/kg PEG-Exa), 0.0002 (5 μmol/kg PEG-Exa), <0.0001 (10 and 15 μmol/kg PEG-Exa). **B,** Median relative body weight of mice over time. Mice (*N* = 6 per group) received a single intraperitoneal dose of vehicle (●), PEG-Exa at 15 μmol/kg (■), 10 μmol/kg (▲), 5 μmol/kg (▼), 2.5 μmol/kg (◆), or PLX038A at 15 μmol/kg (○).

It has been reported that a total dose of 25 and 75 mg (57 and 170 μmol)/kg Exa administered as administered four times at 4-day intervals caused regression of BRCA1-deficient MX-1 TNBC xenografts at 28 days (26). Figure 3 shows the effect of varying single intraperitoneal doses of 2.5 to 15 μmol/kg PEG-Exa 3A on inhibition of growth of the MX-1 TNBC xenografts. As shown, single doses of 10 and 15 μmol/kg PEG-Exa caused complete growth inhibition of tumors without weight loss of hosts for >48 days (Fig. 3). Note that the single 10 μmol/kg PEG-Exa dose is 6- and 18-fold lower than the dose of free Exa required to suppress MX-1 xenografts (26). The growth inhibition for 2.5 μmol/kg PEG-Exa is nearly identical to that of 15 μmol/kg PEG-SN-38 (PLX038A) indicating that PEG-Exa is approximately 6-fold more potent than PLX038A in growth suppression of this tumor**;** the *in vitro* antiproliferative activity of Exa has also been reported to be significantly more potent than SN-38 (5, 27).

### Tumor Inhibition by PEG-Exa Combinations with TLZ or VX970

We measured the effects of a combination of PEG-Exa with the PARPi TLZ and the ATRi VX970 on growth of MX-1 xenografts. TG and TGI were calculated as described previously (25) using the fractional AUC under the tumor volume versus t curve for the treated group in relation to that of the vehicle; that is, TG = [AUC_treatment_/AUC_vehicle_] and TGI = 1 − TG. To assess the interaction of a drug combination, we used an additivity index (28) determined as TG_calc_/TG_obsd_ where TG_calc_ is the product of TG_obsd_ values of the individual drugs and TG_obsd_ is the observed tumor growth in the presence of both drug. Here, an index <1 indicates infra-additive, an index of 1 indicates additivity and an index >1 indicates a supra-additive or synergistic interaction.

**FIGURE 4:**
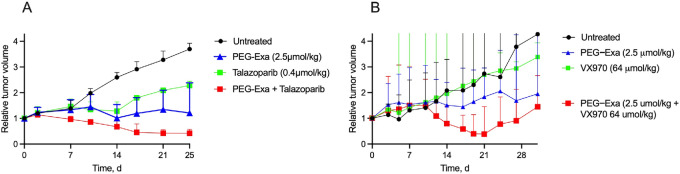
PEG-Exa combinations with TLZ or VX970.  **A,** Median relative tumor volume ± interquartile range versus time for PEG-Exa, TLZ, or a combination. The starting tumor volume for all groups was 156 ± 57 mm^3^. Mice (*N* = 6/group) received a single intraperitoneal dose of vehicle (●), a single intraperitoneal dose of PEG-Exa at 2.5 μmol/kg (▲), QD PO TLZ at 0.4 μmol/kg/day (▲), or a combination of both agents at the same doses (■). *P* values at 21 days from one-way ANOVA analysis versus vehicle were: 0.002 (PEG-Exa), 0.02 (TLZ) <0.0001 (PEG-Exa + TLZ). **B,** Median relative tumor volume ± interquartile range versus time for PEG-Exa, VX970 or a combination. The starting tumor volume for all groups was 130 ± 30 mm^3^. Mice (*N* = 6/group) received a single intraperitoneal dose of vehicle (●), a single intraperitoneal dose of PEG-Exa at 2.5 μmol/kg (▲), orally VX-970 at 64 μmol/kg for 4 consecutive days per week (▲), or a combination of both agents at the same doses (■). The *P* value at 21 days from one-way ANOVA analysis versus vehicle was 0.006 for the PEG-Exa + VX970 group.

Figure 4A shows the tumor growth curves in mice (*n* = 6) treated with 2.5 μmol/kg PEG-Exa as a single intraperitoneal dose, 0.4 μmol (0.15 mg)/kg of TLZ orally every day, and a combination of both. The TG_obsd_ was 0.71 for TLZ and 0.54 for 2.5 μmol/kg PEG-Exa, giving a TG_calc_ for the combination of 38. The TG_obsd_ of the combination was 0.32 so the additivity index was estimated as 1.2 (0.38/0.32), indicating synergy of the two drugs. There was insignificant (<4%) weight loss in treated groups (Supplementary Data; Supplementary Fig. S2A).

Figure 4B shows the tumor growth curves in mice (*n* = 6) treated with 2.5 μmol/kg PEG-Exa as a single intraperitoneal dose, 64 μmol (30 mg)/kg of the ATRi VX970 PO for 4 consecutive days a week (29), and a combination of both; weight loss of mice treated with individual or combined drugs over 30 days was <10% (Supplementary Fig. S2B). The TG_obsd_ was 0.85 for PEG-Exa and 1.04 for VX970 (i.e., TGI = 0), giving a TG_calc_ for the combination of 0.88. The TG_obsd_ of the combination was 0.50, giving an additivity index of 1.7 which indicates strong synergy of the two drugs. Likewise, an additivity index of 1.5 was calculated from results of a similar experiment using the same 2.5 μmol/kg PEG-Exa with a lower dose of 16 μmol/kg VX970 (Supplementary Table S3). Hence, doses of VX970 that do not affect tumor growth strongly act synergistically to enhance the chemotherapeutic activity of PEG-Exa.

## Discussion

We have been developing antigen-independent, long-acting prodrugs of TOP1i with the objective of optimizing DNA-damaging agents for use with DDR deficiencies or with DDR inhibitors. Such agents are differentiated from an ADC TOP1i in that they target tumors with DDR defects rather than antigen-specific tumor types. Synthetic lethality can be achieved when TOP1i are used in DDR-deficient tumors and/or in combination with an appropriate DDRi; the TOP1i creates DNA damage and the DDR defect or DDRi inhibits repair. Because synergy of the TOP1i and a DDRi requires concurrent presence of DNA damage and the DDRi, we posit that a prolonged duration of DNA damage would be permissive of more frequent and/or more intense DDRi dosing than possible with currently available short-acting TOP1i.

We previously reported studies on PLX038, a long-acting prodrug of SN-38, composed of a 4-arm 40 kDa PEG attached to SN-38 by releasable linkers that slowly cleave to release the drug. The prodrug and released SN-38 have long t_1/2_ values of 5 days in humans, approximately 10-fold longer than the SN-38 released from CPT-11. Also, the small 15 nm nanomolecules readily penetrate large pores of tumor vasculature, and accumulate and are retained in the tumor microenvironment for long periods (20). PLX038 is currently being studied in clinical trials (NCT05465941; NCT04209595).

The current study focuses on Exa, another potent inhibitor of TOP1 with potential benefits over CPT-11/SN-38 (5). First, it is even more potent than SN-38 as a TOP1i, which may result in less-off target effects. Second, because it is not extensively metabolized, there should be less interpatient variability than with CPT-11. Finally, Exa does not appear to be a substrate for drug-efflux pumps that may cause resistance to CPT-11/SN-38 (27, 30)

The objective of this work was to develop a long-acting prodrug of Exa that benefits from improved pharmacokinetics and pharmacology. The specific aims were to (i) develop chemistry that enables attachment of Exa to long-lived carriers via β-eliminative linkers, (ii) determine the pharmacokinetics of PEG-Exa in mice, and (iii) ascertain the antitumor activity of the releasable PEG-Exa 3B in mouse xenografts.

To prepare the PEG-Exa conjugates, we coupled Exa with appropriate linker carbonates to provide both stable and β-eliminative releasable amino–linker–Exa intermediates. After attachment of these to PEG_40kDa_ via amides, the conjugate 3B with the stable linker lacking a modulator was completely stable at basic pH for long periods; in contrast, the conjugate with the β-eliminative linker 3A released Exa in a base-catalyzed reaction with a t_1/2_ of 60 hours at pH 7.4. Unlike the large 345 kDa CM-dextran polyalcohol-Exa conjugate, DE-310, 3B does not require cell internalization and proteolytic cleavage to release its Exa payload.

The pharmacokinetics of the PEG_40kDa_-Exa conjugates were studied in the mouse. The stable PEG_40kDa_-Exa 3B shows a renal elimination rate of t_1/2_ approximately 18 hours, similar to that of a typical stable PEG_40kDa_ conjugate in the mouse (19), whereas the releasable PEG_40kDa_-Exa 3A shows a faster apparent elimination t_1/2,β_ of approximately 12 hours. The difference between the rates of 3A and 3B elimination represents the rate of *in vivo* release of Exa from 3A which was equivalent to a t_1/2_ of approximately 40 hours, similar to that obtained *in vitro* (t_1/2_, of 60 hours). The free Exa released from 3A showed a t_1/2_ of 25 hours and represented ≥1,000-fold lower concentration than the prodrug 3A. These parameters are consistent with those reported for the analogous PEG_40kDa_–SN-38 conjugate, PLX038A (22).

We tested the antitumor effects of PEG_40kDa_-Exa against the BRCA1-deficient MX-1 xenograft which is quite sensitive to TOP1i, including free Exa (14) and the long-acting PEG-SN-38 conjugate PLX038A (22). From comparisons of growth curves, we estimate that PEG-Exa is approximately 6-fold more potent than PEG–SN-38 in this xenograft. Remarkably, a single low dose of 10 μmol/kg PEG-Exa—only approximately 0.2 μmol/mouse—caused tumor growth suppression lasting over 40 days.

We pondered why the antitumor effects of a single low dose of PEG_40kDa_-Exa 3A in mouse xenografts are so long lasting. First, the high exposure of released Exa over prolonged periods should have profound antitumor effects—much greater than intermittent dosing of free Exa. Second, tumor cells may become significantly more sensitive to a drug as a function of the time of exposure (31, 32); indeed, the inhibitory potency of Exa increases about 80-fold upon prolonged exposure (30). Here, the lower Exa concentration requirements of an increasingly-sensitive tumor over time would counter the reduced concentration that occurs over time by renal elimination. Finally, large PEGylated prodrugs can accumulate and be retained in tumors, releasing the cargo in the tumor environment over long periods. Indeed, the analogous PEG prodrug of SN-38, PLX038A (20), has a high accumulation of approximately 10% of the initial dose in the MX-1 xenograft and very long efflux t_1/2_ of 17 days.

It has been well established that a combination of a TOP1i and an appropriate DDRi partner can be highly synergistic in many DDR-deficient tumors. An obvious DDRi partner for a long-acting TOP1i would a PARPi, and the BRCA1-deficient MX-1 tumor used here is highly sensitive to both drugs. Indeed, as with PLX038 (28), PEG-Exa is synergistic with the PARPi TLZ. However, despite promising preclinical data such as reported here, TOP1i and PARPi combinations have proven challenging in the clinic where myelosuppression has required large dose reductions of both single agents (3). It has been proposed that modification of the interval between dosing a long-acting, nanomolecular TOP1i and a PARPi might avoid overlapping toxicities (3) and this hypothesis warrants testing in the clinic.

Another candidate DDRi partner for a long-acting TOP1i is an ATRi. Pommier and colleagues identified ATR as a synthetically lethal gene for TOP1is (33), and showed that at nontoxic doses ATRi are strongly synergistic with conventional short-acting TOP1i—including Exa (5, 33). Furthermore, in a phase II trial of relapsed small cell lung cancer, combination of the TOP1i topotecan with the ATRi VX-970 showed a high 36% ORR and acceptable side effects even though administration of both drugs was confined to a 5-day interval of a 21-day cycle (34). We speculate that the prolonged DNA damage expected from a long acting TOP1i in a DDR-deficient tumor would allow more frequent dosing of an ATRi to push tumors beyond their survival threshold.

On the basis of these reports and rationale, we tested the effect of the ATRi VX970 in combination with long-acting PEG-Exa in BRCA-deficient MX-1 xenografts. First, we showed that a relatively high dose of 64 μmol/kg of VX970 for 4 consecutive days a week had no effect on growth of MX-1 xenografts in spite of their BRCA deficiency. Then, we showed that a combination of the same ineffectual dose of VX970 with a single administration of a low 2.5 μmol/kg of PEG-Exa resulted in strong synergy. Hence, the combination of a single low-dose of a long-acting TOP1i with low doses of ATRi are synergistic and synthetically lethal, and should enable a flexible dosing schedule with maximum overlapping exposure of both drugs.

In summary, we developed a long-acting PEGylated prodrug of Exa. A single injection of the prodrug is highly effective in suppressing the BRCA1-deficient MX-1 xenograft for prolonged periods. In addition, PEG-Exa shows synergy with the PARPi TLZ and the ATRi VX970, which provide effective combinations worthy of further studies.

## Supplementary Material

Supplementary Table S1 In vitro t1/2 values for cleavage of PEG-Exa (3A).

Supplementary Table S2 Pharmacokinetic parameters of 3A after 40 μmol/kg IP administration in mice.

Supplementary Table S3 Synergy of PEG-Exa 3A and the ATRi VX-970.

Supplementary Figure S1 C vs t plot of 3A following 40 μmol/kg IP administration in mice.

Supplementary Figure S2 Body weights of mice treated with PEG-Exa combinations with TLZ or VX970.
