# Supplementary material for: A Very Long-acting Exatecan and Its Synergism with DNA Damage Response Inhibitors
Source: Cancer Res Commun. 2023 May 24;3(5):908–16. doi: 10.1158/2767-9764.CRC-22-0517 (PMC10208276; doi:10.1158/2767-9764.CRC-22-0517)
Supplement: Supplementary Table S1 — In vitro t1/2 values for cleavage of PEG-Exa (3A). [file crc-22-0517-s01.docx]

**Table S1.** *In vitro* t_1/2_ values for cleavage of PEG-Exa (**3A**).

| pH, 37° C | Obsd t_1/2_, h | Calc. t_1/2_ at pH 7.5, h |
| --- | --- | --- |
| 9.0 | 1.72 | 67 |
| 8.4 | 4.87 | 53 |
| 7.4 | 51.6 | 57 |
| 5.1 | >99% remaining at 11 d | ND |
|  |  | Ave. 59 ± 7 (SE) |
