# Supplementary material for: A Very Long-acting Exatecan and Its Synergism with DNA Damage Response Inhibitors
Source: Cancer Res Commun. 2023 May 24;3(5):908–16. doi: 10.1158/2767-9764.CRC-22-0517 (PMC10208276; doi:10.1158/2767-9764.CRC-22-0517)
Supplement: Supplementary Table S2 — Pharmacokinetic parameters of 3A after 40 μmol/kg IP administration in mice. [file crc-22-0517-s02.docx]

**Table S2**. Pharmacokinetic parameters of **3A** after 40 μmol/kg IP administration in mice^A^.

^A^ Values for mean ± SEM derived from the data shown in **Fig. S1.** Error values are symmetrical standard errors generated by GraphPad Prism 9.4.1.
