# Supplementary material for: A Very Long-acting Exatecan and Its Synergism with DNA Damage Response Inhibitors
Source: Cancer Res Commun. 2023 May 24;3(5):908–16. doi: 10.1158/2767-9764.CRC-22-0517 (PMC10208276; doi:10.1158/2767-9764.CRC-22-0517)
Supplement: Supplementary Table S3 — Synergy of PEG-Exa 3A and the ATRi VX-970. [file crc-22-0517-s03.docx]

**Table S3.** Synergy of PEG-Exa **3A** and the ATRi VX-970.

| AUC of median MX-1 Tumor volume (Fraction of vehicle control) | | | |
| --- | --- | --- | --- |
|  | VX970  (64 μmol/kg) | VX970  (32 μmol/kg) | VX970  (16 μmol/kg) |
| Vehicle | 1 | 1 | 1 |
| PEG-Exa (**3A** | 0.85 | 0.85 | 0.85 |
| VX970 | 1.04 | 0.96 | 0.98 |
| Observed for combination | 0.50 | 0.70 | 0.51 |
| Predicted for additive combination | 0.88 | 0.81 | 0.83 |
| Predicted/Combined (>1 = synergistic) | 1.7 | 1.2 | 1.6 |
