# Supplementary material for: A Very Long-acting Exatecan and Its Synergism with DNA Damage Response Inhibitors
Source: Cancer Res Commun. 2023 May 24;3(5):908–16. doi: 10.1158/2767-9764.CRC-22-0517 (PMC10208276; doi:10.1158/2767-9764.CRC-22-0517)
Supplement: Supplementary Figure S1 — C vs t plot of 3A following 40 μmol/kg IP administration in mice. [file crc-22-0517-s04.docx]

**Figure S1**. C vs. t plot of **3A** following 40 μmol/kg IP administration in mice.
