# Supplementary material for: A Very Long-acting Exatecan and Its Synergism with DNA Damage Response Inhibitors
Source: Cancer Res Commun. 2023 May 24;3(5):908–16. doi: 10.1158/2767-9764.CRC-22-0517 (PMC10208276; doi:10.1158/2767-9764.CRC-22-0517)
Supplement: Supplementary Figure S2 — Body weights of mice treated with PEG-Exa combinations with TLZ or VX970. [file crc-22-0517-s05.docx]

**Figure S2.** Body weights of mice treated with PEG-Exa combinations with TLZ or VX970. A) Median relative body weights vs. time for PEG-Exa, TLZ or a combination. Mice (N = 5-to 6 per group) received a single IP dose of vehicle (●), a single IP dose of PEG~Exa at 2.5 µmol/kg (▲), QD PO TLZ at 0.4 µmol/kg/d (▲), or a combination of both agents at the same doses (■). B) Median relative body weoghts vs. time for PEG-Exa, VX970 or a combination. Mice (N = 5- to 6 per group) received a single IP dose of vehicle (●), a single IP dose of PEG~Exa at 2.5 µmol/kg (▲), a single IV dose of exatecan at 28 µmol/kg (▼), PO VX-970 at 64 µmol/kg for four consecutive days per week (▲), or a combination of both agents at the same doses (■).
